# Supplementary material for: Longitudinal evaluation of fecal microbiota transplantation for ameliorating calf diarrhea and improving growth performance
Source: Nat Commun. 2021 Jan 8;12:161. doi: 10.1038/s41467-020-20389-5 (PMC7794225; doi:10.1038/s41467-020-20389-5)
Supplement: Supplementary file 1 — Supplementary Information [file 41467_2020_20389_MOESM1_ESM.pdf]

Supplementary Information

Longitudinal evaluation of fecal microbiota transplantation for ameliorating calf diarrhea and improving growth performance

Hyun Sik Kim[1], Tae Woong Whon[1,3], Hojun Sung[1], Yun-Seok Jeong[1], Eun Sung Jung[2], Na-Ri Shin[1,4], Dong-Wook Hyun[1], Pil Soo Kim[1], June-Young Lee[1], Choong Hwan Lee[2], and Jin-Woo Bae[1]*

[1]Department of Life and Nanopharmaceutical Sciences and Department of Biology, Kyung Hee University, Seoul 02447, Republic of Korea

[2]Department of Bioscience and Biotechnology, Konkuk University, Seoul 05029, Republic of Korea

[3]Present address: Microbiology and Functionality Research Group, World Institute of Kimchi, Gwangju 61755, Republic of Korea

[4]Present address: Biological Resource Center, Korea Research Institute of Bioscience and Biotechnology, Jeongeup-si, Jeollabuk-do 56212, Republic of Korea

These authors contributed equally: Hyun Sik Kim, Tae Woong Whon

*e-mail: baejw@khu.ac.kr

Supplementary Information includes:

- Supplementary Figures 1 to 9
- Supplementary Tables 1 to 7

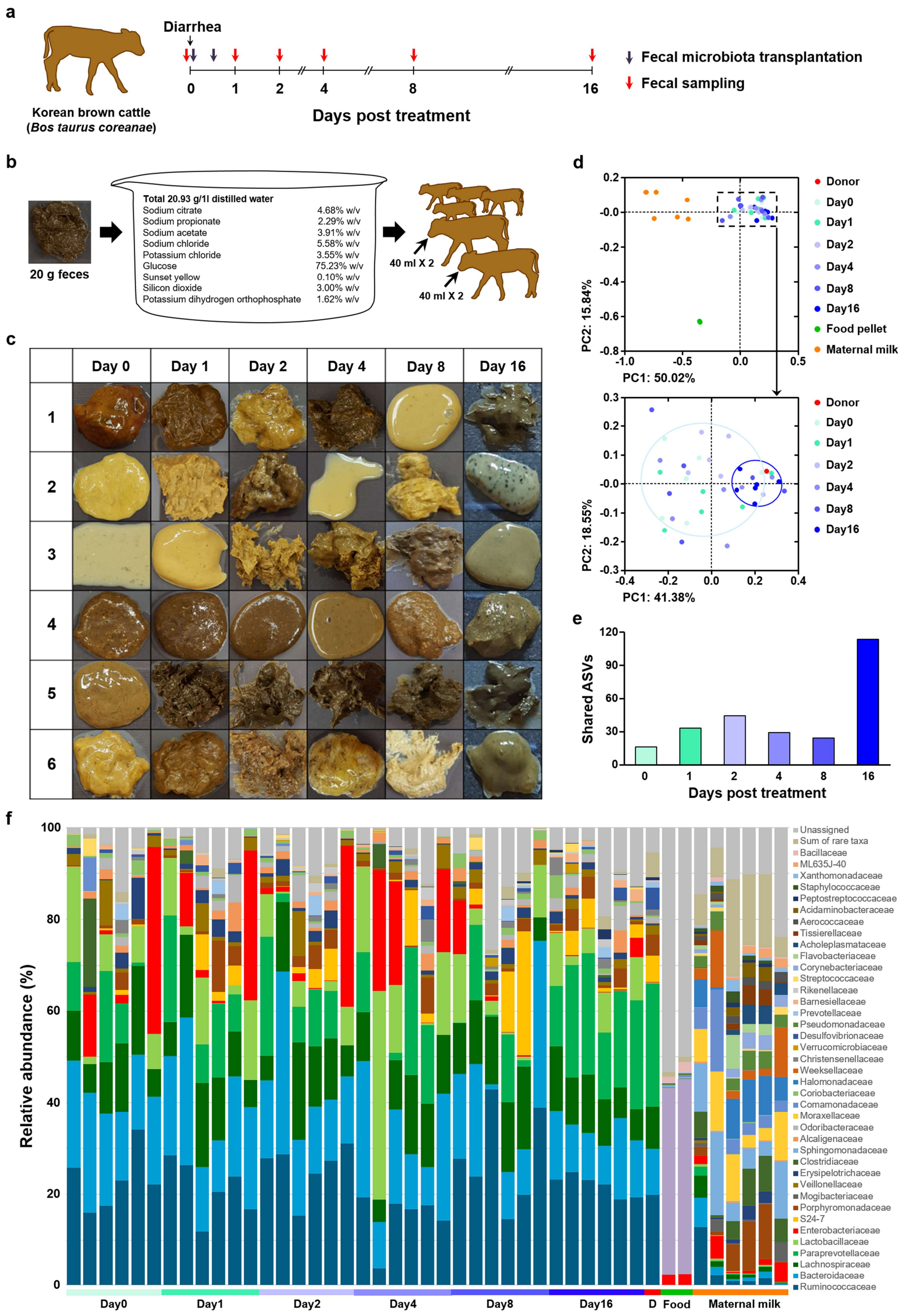
a
Diarrhea
Fecal microbiota transplantation
Fecal sampling
0 1 2 4 8 16
Days post treatment
Korean brown cattle
(Bos taurus coreanae)
b
20 g feces
Total 20.93 g/1l distilled water
Sodium citrate 4.68% w/v
Sodium propionate 2.29% w/v
Sodium acetate 3.91% w/v
Sodium chloride 5.58% w/v
Potassium chloride 3.55% w/v
Glucose 75.23% w/v
Sunset yellow 0.10% w/v
Silicon dioxide 3.00% w/v
Potassium dihydrogen orthophosphate 1.62% w/v
40 ml X 2
40 ml X 2
c
Day 0
Day 1
Day 2
Day 4
Day 8
Day 16
1
2
3
4
5
6
d
PC1: 50.02%
PC2: 15.84%
Donor
Day0
Day1
Day2
Day4
Day8
Day16
Food pellet
Maternal milk
PC1: 41.38%
PC2: 18.55%
e
Shared ASVs
Days post treatment
f
Relative abundance (%)
Unassigned
Sum of rare taxa
Bacillaceae
ML635J-40
Xanthomonadaceae
Staphylococcaceae
Peptostreptococcaceae
Acidaminobacteraceae
Aerococcaceae
Tissierellaceae
Acholeplasmataceae
Flavobacteriaceae
Corynebacteriaceae
Streptococcaceae
Rikenellaceae
Barnesiellaceae
Prevotellaceae
Pseudomonadaceae
Desulfovibrionaceae
Verrucomicrobiaceae
Christensenellaceae
Weeksellaceae
Halomonadaceae
Coriobacteriaceae
Comamonadaceae
Moraxellaceae
Odoribacteraceae
Alcaligenaceae
Sphingomonadaceae
Clostridiaceae
Erysipelotrichaceae
Veillonellaceae
Mogibacteriaceae
Porphyromonadaceae
S24-7
Enterobacteriaceae
Lactobacillaceae
Paraprevotellaceae
Lachnospiraceae
Bacteroidaceae
Ruminococcaceae
Day0
Day1
Day2
Day4
Day8
Day16
D
Food
Maternal milk

Supplementary Fig. 1 Details of the preliminary trial conducted to test the safety of oral FMT in calves

a Design of the preliminary FMT trial in calves. **b** Procedures for the preliminary FMT trial. Twenty grams of feces from one healthy donor calf was homogenized and diluted in buffer solution composed of 4% saline [4.68% sodium citrate (w/v), 2.29% sodium propionate (w/v), 3.92% sodium acetate (w/v), 5.59% sodium chloride (w/v), 3.56% potassium chloride (w/v), 75.23% glucose (w/v), 0.1% sunset yellow (w/v), 3.0% silicon dioxide (w/v), and 1.62% potassium dihydrogen orthophosphate (w/v)] and 10% glycerol cryoprotectant. Six recipient calves were administered 40 ml fecal suspension (0.0005 g/ml final concentration) twice daily. **c** Images of the feces collected per rectum from recipient calves 0, 1, 2, 4, 8, and 16 days after the treatment commenced. **d** PCoA, based on the weighted UniFrac distance matrix, of the bacterial 16S rRNA gene sequence data for the feed pellet, maternal milk, and fecal samples 0, 2, 4, 8, 16, 32, and 48 days after the start of treatment. **e** The number of ASVs shared between the feces of recipient calves and the healthy donor calf. **f** The relative abundances of the abundant bacterial families (>0.5% of the mean abundance) shown by metataxonomic analysis of fecal samples are displayed as bar graphs. FMT, fecal microbiota transplantation. Source data are provided as a Source Data file.

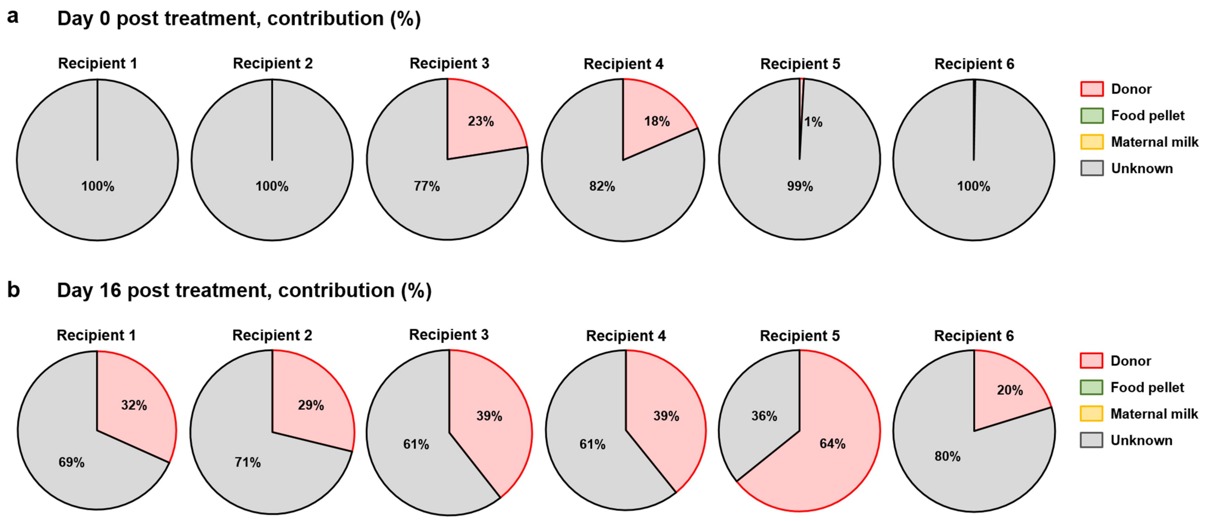

Supplementary Fig. 2 Estimation of the contribution of each source of bacteria to the microbial communities of the recipient calves in the preliminary trial

a–b Results of SourceTracker analysis, showing the mean contributions of each source of bacteria to the bacterial communities of the recipient calves **(a)** on day 0 and **(b)** day 16 after the start of treatment. The sources of the bacteria were the gut microbiota of donor feces, food pellets, and maternal milk. Related to Supplementary Fig. 1e. Source data are provided as a Source Data file.

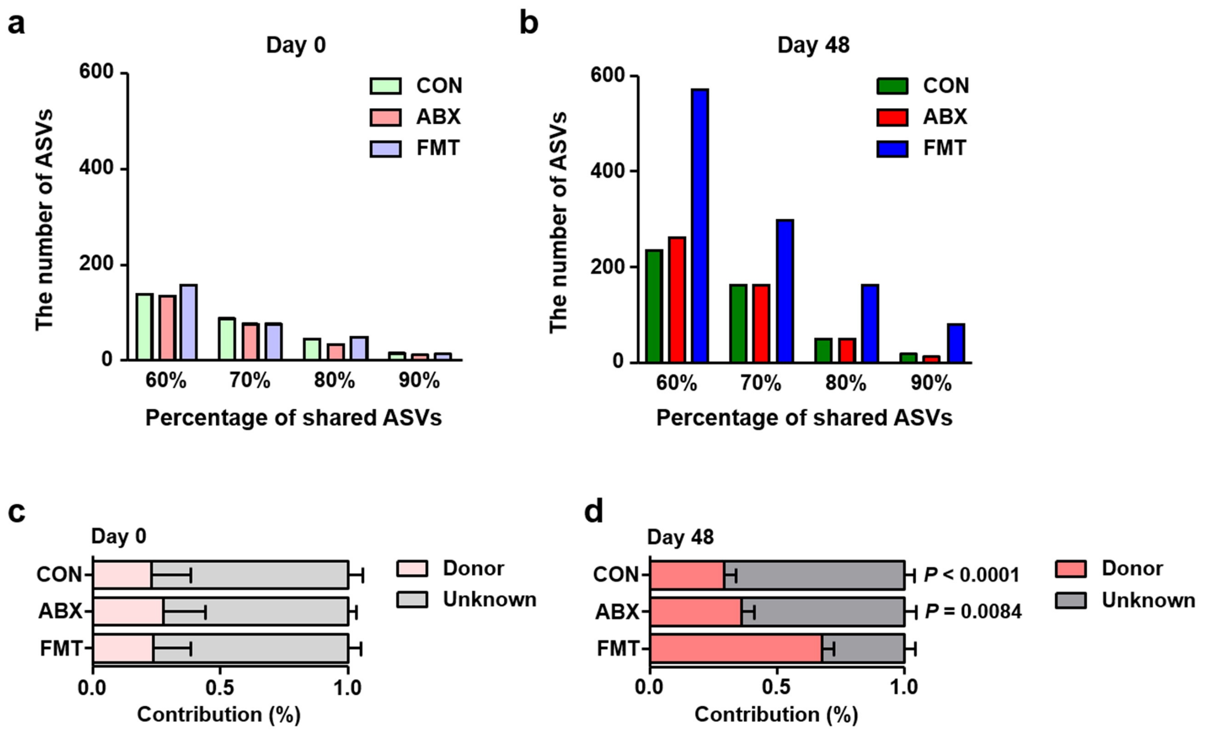

Supplementary Fig. 3 Contributions of donor feces to the bacterial communities in the CON, ABX, and FMT calves

a–b The number of ASVs shared between the feces of healthy donor calves and the CON, ABX, and FMT calves on **(a)** day 0 and **(b)** day 48 after the start of treatment. The percentages of ASVs shared are shown. **c–d** Results of SourceTracker analysis showing the mean contributions of donor feces to the bacterial communities in the CON (n = 14), ABX (n = 23), and FMT (n = 20) calves on **(c)** day 0 and **(d)** day 48. Source: gut microbiome of donor calves; sinks: CON, ABX, and FMT calves. CON, control; ABX, antibiotic; FMT, fecal microbiota transplantation. Data are shown as mean ± SEM. The P-values were determined using the Mann–Whitney U-test (two-tailed). Related to Fig. 2l. Source data are provided as a Source Data file.

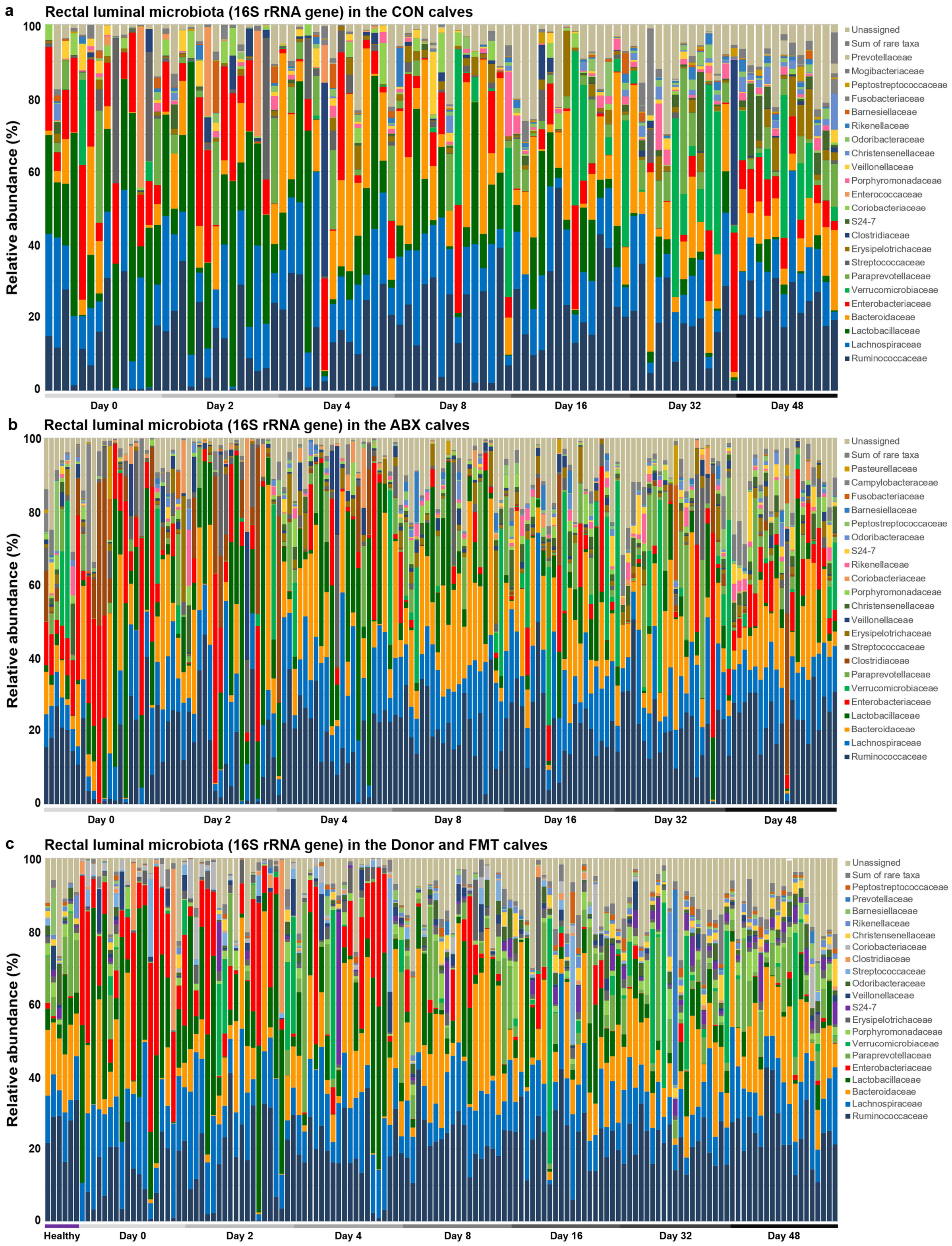

Supplementary Fig. 4 Relative abundances of the intestinal microbial taxa in the groups during the of the trial

a–**c** The relative abundances of the abundant bacterial families (>0.5% of the mean abundance) shown by metataxonomic analysis of CON **(a)**, ABX **(b)**, and FMT **(c)** fecal samples are

displayed as bar graphs. CON, control; ABX, antibiotic; FMT, fecal microbiota transplantation. Related to Fig. 2a–c. Source data are provided as a Source Data file.

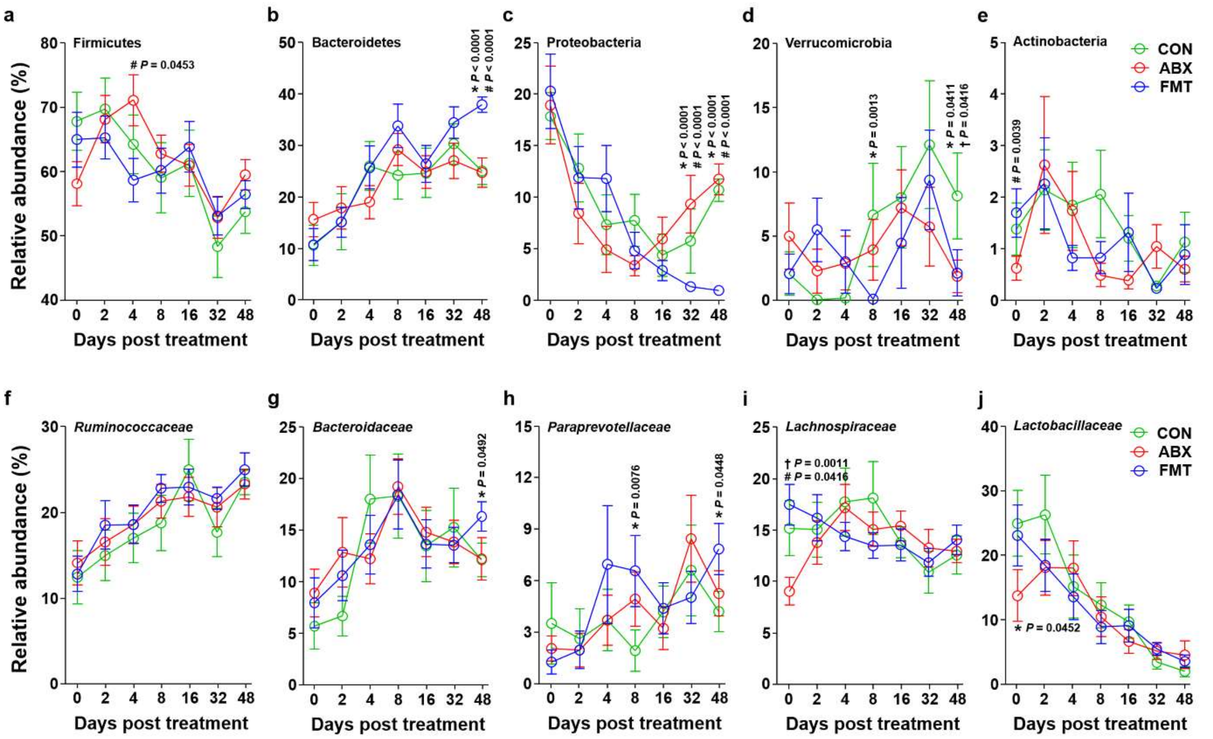

Supplementary Fig. 5 Changes in the relative abundances of the predominant bacterial phyla and families during the trial

a–e The abundances of the phyla **(a)** Firmicutes, **(b)** Bacteroidetes, **(c)** Proteobacteria, **(d)** Verrucomicrobia and **(e)** Actinobacteria are shown, according to treatment group (CON, n = 14; ABX, n = 23; FMT, n = 20). **f–j** The abundances of the families **(f)** *Ruminococcaceae*, **(g)** *Bacteroidaceae*, **(h)** *Paraprevotellaceae*, **(i)** *Lachnospiraceae* and **(j)** *Lactobacillaceae* are shown, according to treatment group (CON, n = 14; ABX, n = 23; FMT, n = 20). Data are shown as mean ± SEM. The *P*-values were determined using the Mann–Whitney *U*-test (two-tailed). *Comparison of the CON and FMT groups; #comparison of the ABX and FMT groups; and †comparison of the CON and ABX groups. CON, control; ABX, antibiotic; FMT, fecal microbiota transplantation. Related to Fig. 3e. Source data are provided as a Source Data file.

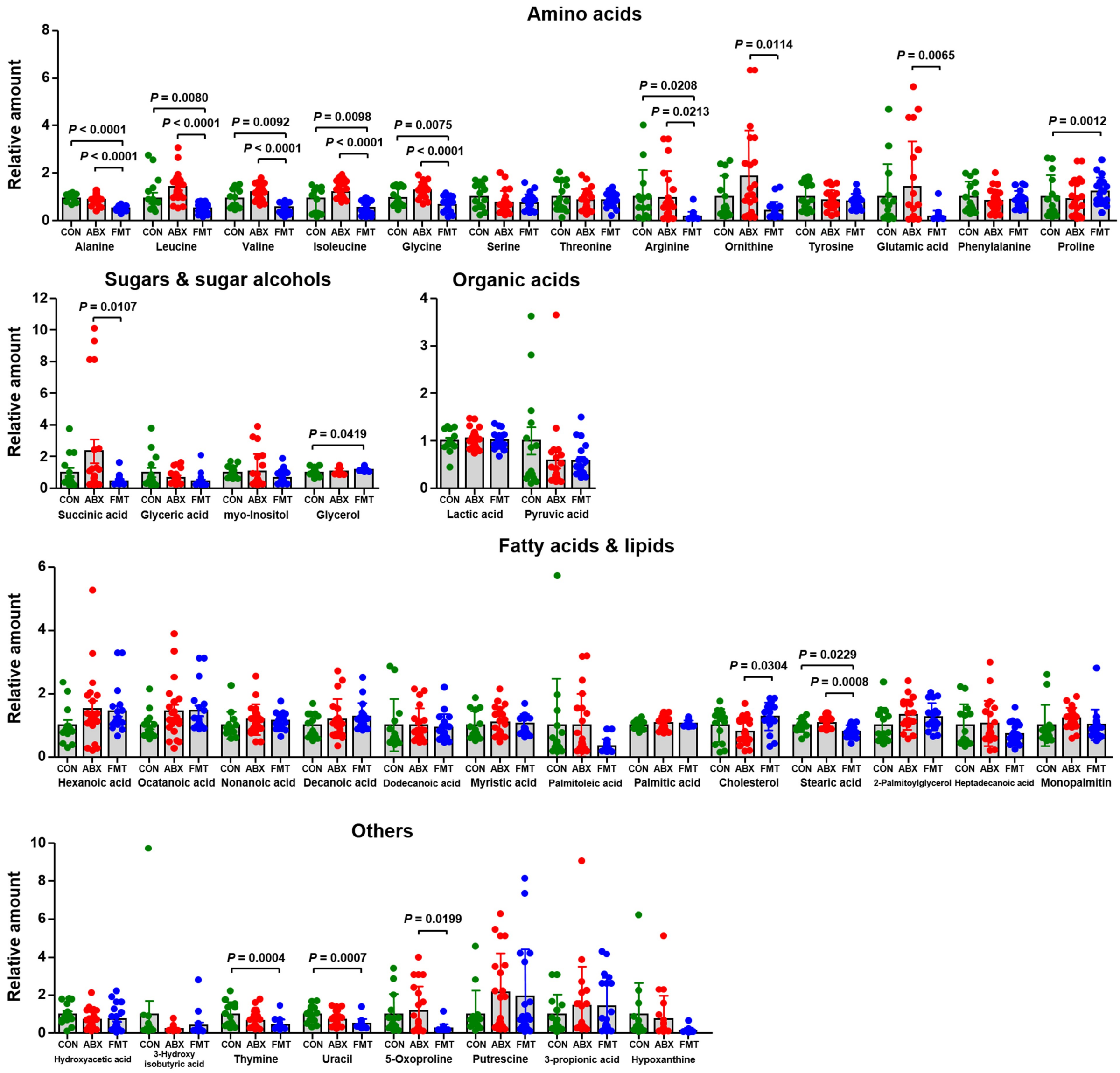

Supplementary Fig. 6 Relative concentrations of fecal metabolites 48 days after the start of treatment

The relative concentrations of the fecal metabolites are displayed as bar chart and dot plots (CON, n = 14; ABX, n = 23; FMT, n = 20). Data are shown as mean ± SEM. The P-values were determined using the Mann–Whitney U-test (two-tailed). Related to Fig. 4b–d. Source data are provided as a Source Data file.

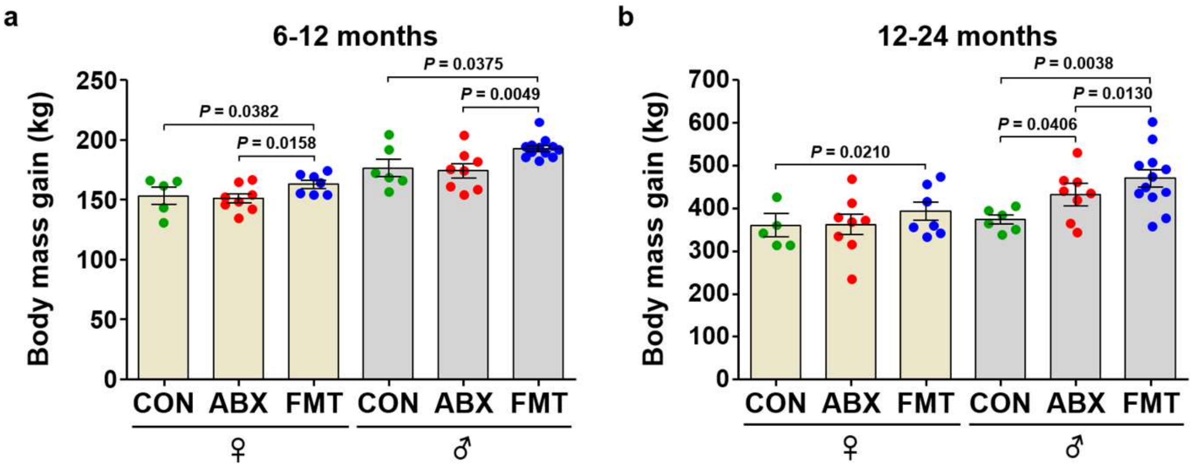

Supplementary Fig. 7 Body mass gain of the cattle

Body mass gain of the cattle (*n* = 50) during their 6 months (**a**, from 6-month-old to 12-month-old) and 12 months (**b**, from 12-months-old to 24-months-old) of life. Bar graphs and dot plots are colored according to sex and treatment. Data are shown as mean ± SEM. The *P*-values were determined using the Mann–Whitney *U*-test (two-tailed). CON, control; ABX, antibiotic; FMT, fecal microbiota transplantation. Related to Fig. 5a–d. Source data are provided as a Source Data file.

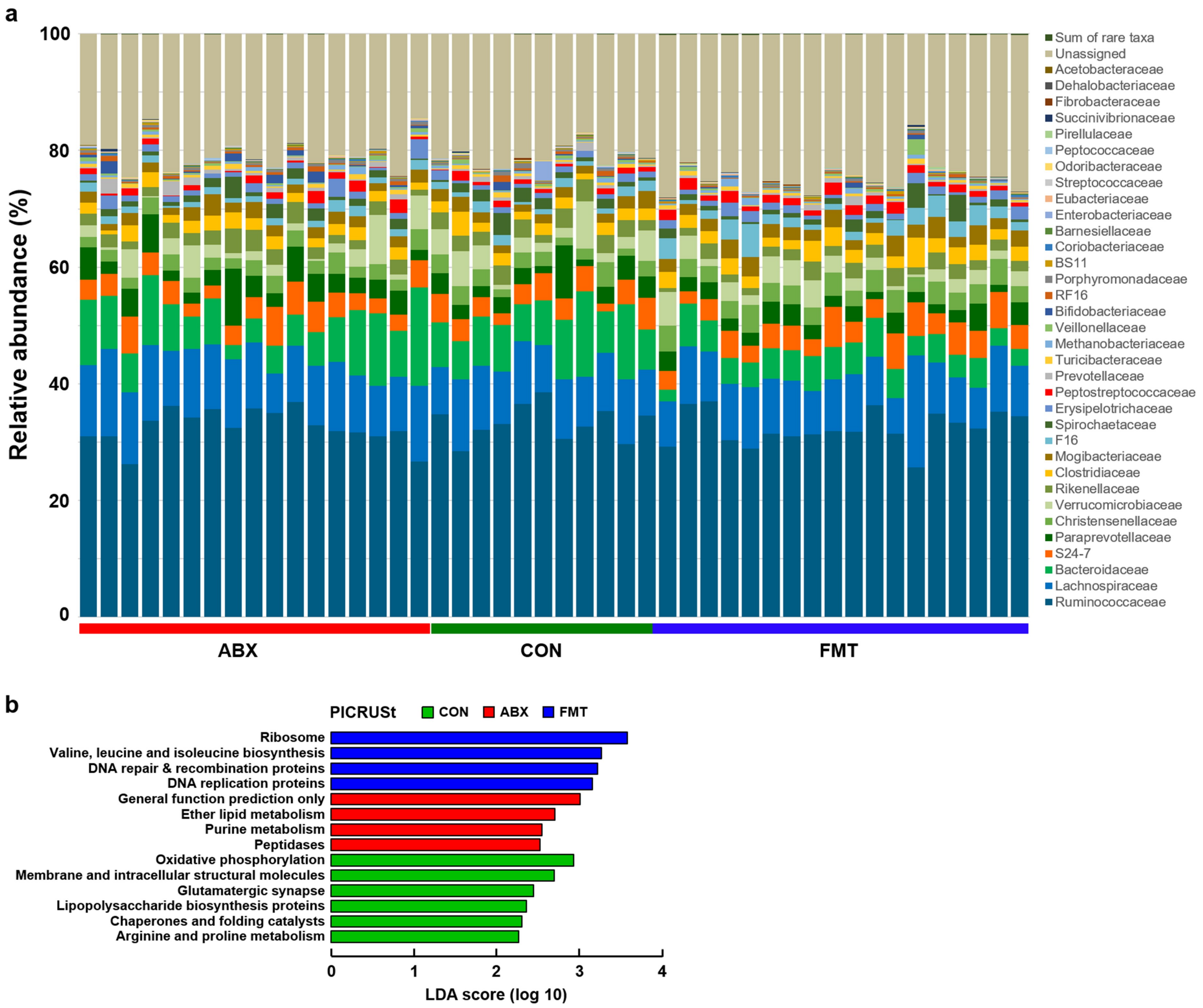

Supplementary Fig. 8 Relative abundances of the intestinal microbiota in 12-month-old CON, ABX, and FMT cattle

a The relative abundances of the abundant bacterial families (>0.5% of the mean abundance) shown by the metataxonomic analysis of fecal samples are displayed as a bar graph. **b** The predicted functional abundance of the rectal microbiota in 12-month-old CON, ABX, and FMT cattle, as determined using LEfSe and PICRUSt, respectively. The abundances are represented by LDA scores (>2.0). CON, control; ABX, antibiotic; FMT, fecal microbiota transplantation. Related to Fig. 5e–i. Source data are provided as a Source Data file.

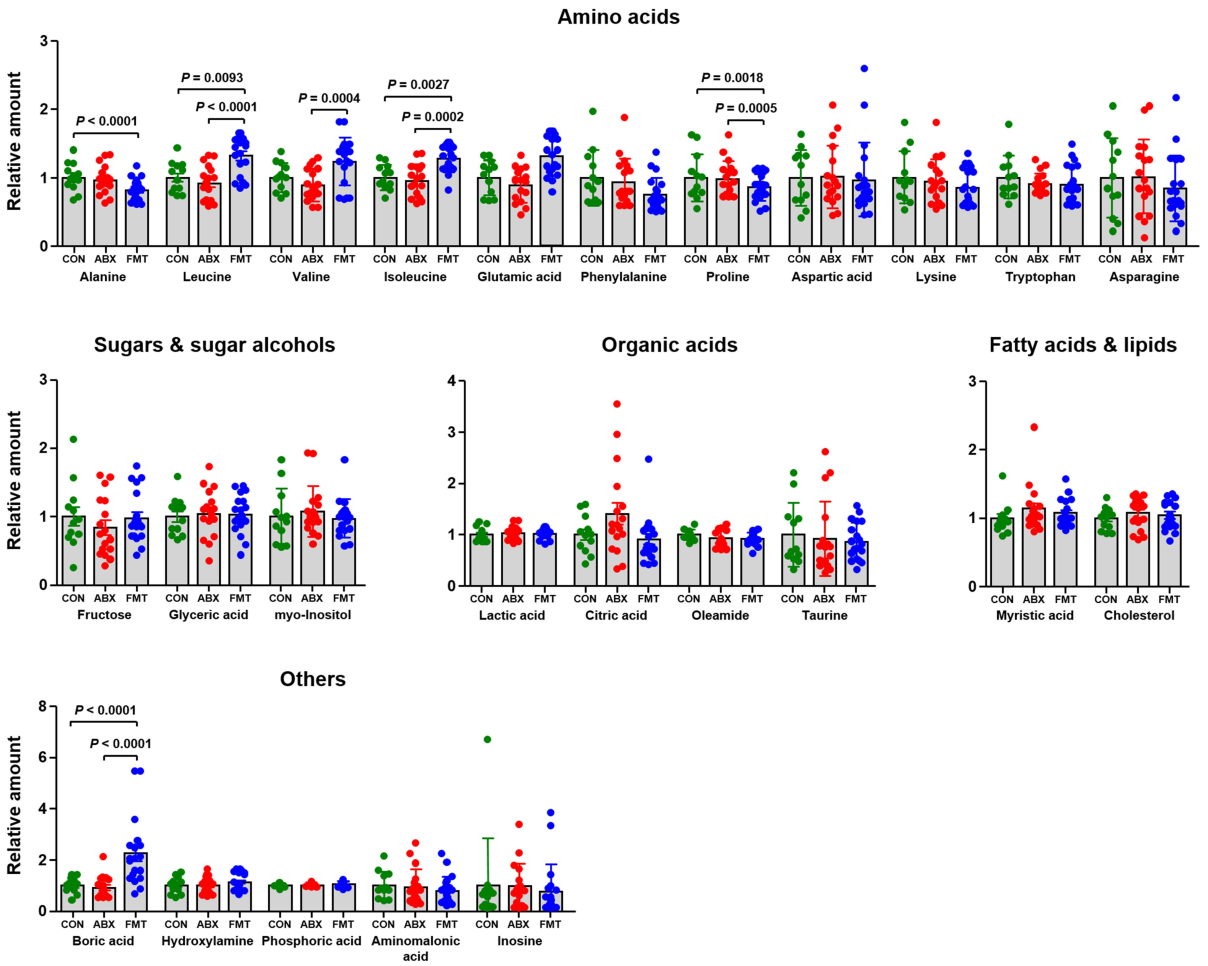

Supplementary Fig. 9 Relative concentrations of serum metabolites in 12-month-old cattle

The relative concentrations of serum metabolites are displayed as bar chart and dot plots (CON, n = 11; ABX, n = 19; FMT, n = 20). Data are shown as mean ± SEM. The P-values were determined using the Mann–Whitney U-test (two-tailed). Related to Fig. 6a–b. Source data are provided as a Source Data file.

Supplementary Table 1 Description of the samples collected during the preliminary trial

Sample ID	Sample	#Sample	Sex	Age (days)	Analysis
Donor	Feces	1	M	24	16S rRNA sequencing
Recipient 1	Feces	6	M	14–30	16S rRNA sequencing
Recipient 2	Feces	6	F	12–28	16S rRNA sequencing
Recipient 3	Feces	6	F	19–35	16S rRNA sequencing
Recipient 4	Feces	6	F	22–38	16S rRNA sequencing
Recipient 5	Feces	6	M	10–26	16S rRNA sequencing
Recipient 6	Feces	6	M	9–25	16S rRNA sequencing
Mother 1	Milk	1	F	N/A	16S rRNA sequencing
Mother 2	Milk	1	F	N/A	16S rRNA sequencing
Mother 3	Milk	1	F	N/A	16S rRNA sequencing
Mother 4	Milk	1	F	N/A	16S rRNA sequencing
Mother 5	Milk	1	F	N/A	16S rRNA sequencing
Mother 6	Milk	1	F	N/A	16S rRNA sequencing
Feed 1	Feed	1	N/A	N/A	16S rRNA sequencing
Feed 2	Feed	1	N/A	N/A	16S rRNA sequencing

Abbreviations: M, male; F, female; #, number of samples; N/A, not applicable.

Supplementary Table 2 Details of the CON samples collected during the validation trial

Sample ID	Sample (#)		Sex	Age (days)	Remission	Death	Analysis
CON1	Feces (8)	Serum (2)	F	19	N	N	16S rRNA sequencing, Metabolomics
CON2	Feces (8)	Serum (2)	M	10	N	N	16S rRNA sequencing, Metabolomics
CON3	Feces (8)	Serum (2)	F	11	N	N	16S rRNA sequencing, Metabolomics
CON4	Feces (8)	Serum (2)	M	21	N	N	16S rRNA sequencing, Metabolomics
CON5	Feces (8)	Serum (2)	M	16	Y	N	16S rRNA sequencing, Metabolomics
CON6	Feces (8)	Serum (2)	M	17	Y	N	16S rRNA sequencing, Metabolomics
CON7	Feces (8)	Serum (2)	F	24	Y	N	16S rRNA sequencing, Metabolomics
CON8	Feces (8)	Serum (2)	F	28	N	N	16S rRNA sequencing, Metabolomics
CON9	Feces (7)	-	M	13	N	Y	16S rRNA sequencing
CON10	Feces (8)	Serum (2)	M	14	Y	N	16S rRNA sequencing, Metabolomics
CON11	Feces (8)	Serum (2)	F	20	N	N	16S rRNA sequencing, Metabolomics
CON12	Feces (8)	Serum (2)	F	17	N	N	16S rRNA sequencing, Metabolomics
CON13	Feces (5)	-	F	27	N	Y	16S rRNA sequencing
CON14	Feces (8)	Serum (2)	M	15	Y	N	16S rRNA sequencing, Metabolomics
*P-value for FMT *vs.* CON			0.583	0.699	0.0003	0.069	-
*P-value for FMT *vs.* ABX			0.831	0.549	< 0.0001	0.016	-

Abbreviations: M, male; F, female; Y, yes; N, no; #, number of samples.

*Data were analyzed using the Mann–Whitney U-test (two-tailed).

Supplementary Table 3 Details of the ABX samples collected during the validation trial

Sample ID	Sample (#)		Sex	Age (days)	Remission	Death	Analysis
ABX1	Feces (7)	-	F	15	N	Y	16S rRNA sequencing
ABX2	Feces (7)	-	M	15	N	Y	16S rRNA sequencing
ABX3	Feces (7)	-	M	11	N	Y	16S rRNA sequencing
ABX4	Feces (8)	Serum (2)	M	22	N	N	16S rRNA sequencing, Metabolomics
ABX5	Feces (8)	Serum (2)	M	20	N	N	16S rRNA sequencing, Metabolomics
ABX6	Feces (8)	Serum (2)	F	19	N	N	16S rRNA sequencing, Metabolomics
ABX7	Feces (3)	-	F	18	N	Y	16S rRNA sequencing
ABX8	Feces (8)	Serum (2)	M	8	N	N	16S rRNA sequencing, Metabolomics
ABX9	Feces (8)	Serum (2)	F	11	N	N	16S rRNA sequencing, Metabolomics
ABX10	Feces (8)	Serum (2)	M	29	N	N	16S rRNA sequencing, Metabolomics
ABX11	Feces (8)	Serum (2)	M	26	N	N	16S rRNA sequencing, Metabolomics
ABX12	Feces (8)	Serum (2)	F	13	N	N	16S rRNA sequencing, Metabolomics
ABX13	Feces (8)	Serum (2)	F	15	Y	N	16S rRNA sequencing, Metabolomics
ABX14	Feces (8)	Serum (2)	F	16	N	N	16S rRNA sequencing, Metabolomics
ABX15	Feces (8)	Serum (2)	M	13	Y	N	16S rRNA sequencing, Metabolomics
ABX16	Feces (8)	Serum (2)	F	15	Y	N	16S rRNA sequencing, Metabolomics
ABX17	Feces (8)	Serum (2)	M	11	Y	N	16S rRNA sequencing, Metabolomics
ABX18	Feces (8)	Serum (2)	M	21	Y	N	16S rRNA sequencing, Metabolomics
ABX19	Feces (8)	Serum (2)	F	25	Y	N	16S rRNA sequencing, Metabolomics
ABX20	Feces (8)	Serum (2)	M	18	N	N	16S rRNA sequencing, Metabolomics
ABX21	Feces (8)	Serum (2)	F	22	N	N	16S rRNA sequencing, Metabolomics
ABX22	Feces (8)	Serum (2)	M	20	N	N	16S rRNA sequencing, Metabolomics
ABX23	Feces (8)	Serum (2)	M	24	N	N	16S rRNA sequencing, Metabolomics
*P-value for FMT *vs.* CON			0.583	0.699	0.0003	0.069	-
*P-value for FMT *vs.* ABX			0.831	0.549	< 0.0001	0.016	-

Abbreviations: M, male; F, female; Y, yes; N, no; #, number of samples.

*Data were analyzed using the Mann–Whitney U-test (two-tailed).

Supplementary Table 4 Details of the FMT samples collected during the validation trial

Sample ID	Sample (#)		Sex	Age (days)	Remission	Death	Analysis
Donor 1	Feces (1)	-	M	34	-	-	16S rRNA sequencing
Donor 2	Feces (1)	-	F	31	-	-	16S rRNA sequencing
Donor 3	Feces (1)	-	M	36	-	-	16S rRNA sequencing
Donor 4	Feces (1)	-	F	48	-	-	16S rRNA sequencing
Donor 5	Feces (1)	-	M	34	-	-	16S rRNA sequencing
Donor 6	Feces (1)	-	M	40	-	-	16S rRNA sequencing
FMT1	Feces (8)	Serum (2)	M	15	Y	N	16S rRNA sequencing, Metabolomics
FMT2	Feces (8)	Serum (2)	M	11	Y	N	16S rRNA sequencing, Metabolomics
FMT3	Feces (8)	Serum (2)	M	21	Y	N	16S rRNA sequencing, Metabolomics
FMT4	Feces (8)	Serum (2)	F	25	Y	N	16S rRNA sequencing, Metabolomics
FMT5	Feces (8)	Serum (2)	F	16	Y	N	16S rRNA sequencing, Metabolomics
FMT6	Feces (8)	Serum (2)	M	16	Y	N	16S rRNA sequencing, Metabolomics
FMT7	Feces (8)	Serum (2)	F	17	Y	N	16S rRNA sequencing, Metabolomics
FMT8	Feces (8)	Serum (2)	M	28	Y	N	16S rRNA sequencing, Metabolomics
FMT9	Feces (8)	Serum (2)	M	24	Y	N	16S rRNA sequencing, Metabolomics
FMT10	Feces (8)	Serum (2)	F	20	Y	N	16S rRNA sequencing, Metabolomics
FMT11	Feces (8)	Serum (2)	F	9	Y	N	16S rRNA sequencing, Metabolomics
FMT12	Feces (8)	Serum (2)	M	16	Y	N	16S rRNA sequencing, Metabolomics
FMT13	Feces (8)	Serum (2)	F	15	N	N	16S rRNA sequencing, Metabolomics
FMT14	Feces (8)	Serum (2)	M	23	Y	N	16S rRNA sequencing, Metabolomics
FMT15	Feces (8)	-	M	19	Y	N	16S rRNA sequencing, Metabolomics
FMT16	Feces (8)	Serum (2)	M	21	Y	N	16S rRNA sequencing, Metabolomics
FMT17	Feces (8)	Serum (2)	F	27	Y	N	16S rRNA sequencing, Metabolomics
FMT18	Feces (8)	Serum (2)	M	17	Y	N	16S rRNA sequencing, Metabolomics
FMT19	Feces (8)	Serum (2)	M	10	Y	N	16S rRNA sequencing, Metabolomics
FMT20	Feces (8)	Serum (2)	F	21	Y	N	16S rRNA sequencing, Metabolomics
*P-value for FMT *vs.* CON			0.583	0.699	0.0003	0.069	-
*P-value for FMT *vs.* ABX			0.831	0.549	< 0.0001	0.016	-

Abbreviations: M, male; F, female; Y, yes; N, no; #, number of samples.

*Data were analyzed using the Mann–Whitney U-test (two-tailed).

Supplementary Table 5 The composition of the diets

Dietary component	Ingredients	Proportion (%)			
		Calf (2–6 months)	Juvenile (7–17 months)	Postpubescent (18–24 months)	Adult mother
Solid feed	Corn	-	5	15	4
	Concentrated feed	10	10	10	10
	Wheat	10	10	10	10
	Brewers' grain	10	10	10	10
	Corn Gluten Feed	-	5	6	5
	Wheat gluten	-	5	5	5
	Rice bran	-	4	5	4
	Soybean curd residue	3	5	5	5
	Oil cake	2	4	4	6
	Soybean meal	5	5	2	-
	Solid feed total	40	63	72	75
Roughage	Timothy	25	11	-	-
	Alfalfa	30	5	-	-
	Sudan grass	-	10	10	15
	Annual Ryegrass	-	-	7	16
	Roughage total	55	26	17	13
Others	Water	4.7	10.8	10.9	9.8
	Vitamin (PK-BV3)	0.3	0.2	0.1	0.2
		100	100	100	100

Supplementary Table 6 Diagnostic multiplex PCR findings in fecal samples from apparently healthy donor calves

Host		Donor																			
		1	2	3	4	5	6	7	8	9	10	11	12	13	14	15	16	17	18	19	20
**Viruses**	Group A rotavirus																		+		
	Group A rotavirus																				
	Group B rotavirus		+																		
	Group C rotavirus		+		+	+	+		+									+			
	Bovine coronavirus											+									
	Bovine coronavirus																				
	Bovine torovirus																				
	Bovine torovirus																				
	Bovine norovirus					+															
	Bovine enteric Nebraska-like calicivirus																				
	Bovine nebovirus																				
	Bovine viral diarrhea virus																+				
	Bovine viral diarrhea virus															+	+				
	Bovine viral diarrhea virus															+	+				
**Bacteria**	*Clostridium perfringens*									+											
	Clostridium perfringens									+											
	Salmonella enterica																				
	Salmonella enterica typhimurium																				
	Enterotoxigenic *Escherichia coli*																			+	
	Shigatoxigenic *Escherichia coli*																				+
	Shigatoxigenic *Escherichia coli*																				+
	Shigatoxigenic *Escherichia coli*																				
	Enterohemorrhagic *Escherichia coli*																				
**Protozoa**	*Eimeria zuernii*										+										

Abbreviations: dsRNA segment 6, double-stranded RNA genome segment 6; VP, viral protein; N, nucleocapsid protein; S, S glycoprotein; M, membrane protein; RdRp, RNA-dependent RNA polymerase; MCP, major capsid protein; BstEII, restriction enzyme BstEII; *sefB*, chaperone protein SefB coding gene; *rfbJ*, CDP-abequose synthase coding gene; *K99*, K99 region 1 gene; *stx1*, shiga toxin type 1; *stx2*, shiga toxin type 2; *eaeA*, enterohemorrhagic *E. coli* O157:H7-specific intimin; *hlyA*, plasmid-encoded enterohemolysin; ITS-1, internal transcribed spacer 1 region of ribosomal RNA gene.

Supplementary Table 7 Primer sets used for the diagnostic multiplex PCR assay

Host		Target gene	Sequence (5' to 3')		Reference
			Forward	Reverse	
**Viruses**	Group A rotavirus	dsRNA segment 6	GGCTTTTAAACGAAGTCTTC	GGTCACATCCTCTCACTACG	1
	Group A rotavirus	VP7	GCCTTTAAAAGCGAGAATTT	GGTCACATCATACAAYTC TA	2
	Group B rotavirus	VP7	GGAAATAATCAGAGATG	CTACTCGTTTGGCTCCCTCC	3
	Group C rotavirus	VP6	TCAAGAAATGGWATGCAACC	CATAGCMGCTGGTCTWATCA	4
	Bovine coronavirus	N	GCAATCCAGTAGTAGAGCGT	CTTAGTGGCATCCTTGCCAA	5
	Bovine coronavirus	S	ATGTTTTTGATACTTTTAATTTCC	ACACCAGTAGATGGTGCTAT	4
	Bovine torovirus	M	TTCTTACTACACTTTTTGGA	ACTCAAACTTAACACTAG AC	4
	Bovine torovirus	N	TAATGGCACTGAAGACTC	ACATAACATCTTACATGG	6
	Bovine norovirus	RdRp	AGTTAYTTTTCCTTYTAYGGBGA	AGTGTCTCTGTCAGTCATCTTCAT	7
	Bovine enteric Nebraska-like calicivirus	RdRp-MCP	TTTCTAACYTATGGGGAYGAYG	GTCACTCATGTTTCCTTCTCTAAT	7
	Bovine nebovirus	Capsid	CCACCATTATCACCAAATTGC	CATAATCAGAATAGAAGGCGC	8
	Bovine viral diarrhea virus	BstEII	GATTTCAAGGGGACTTTTTT	ACATCTCCTACTAAGTAGTA	9
	Bovine viral diarrhea virus	BVDV1 genotype	GTAGTCGTCAGTGGTTCG	GCCATGTACAGCAGAGAT	9
	Bovine viral diarrhea virus	Polyprotein	ACAAACATGGTTGGTGCAACTGGT	CAGACATATTTGCCTAGGTTCCA	10
**Bacteria**	*Clostridium perfringens*	16S rRNA gene	AAAGATGGCATCATCATTCAAC	TACCGTCATTATCTTCCCCAAA	11
	Clostridium perfringens	*alpha-toxin* genes	GCTAATGTTACTGCCGTTGACC	TCTGATACATCGTGTAAG	11
	Salmonella enterica	*sefb*	AGATTGGGCACTACACGTGT	TGTACTCCACCAGGTAATTG	12
	Salmonella enterica typhimurium	*rfbJ*	CCAGCACCAGTTCCAACTTGATAC	GGCTTCCGGCTTTATTGGTAAGCA	13
	Enterotoxigenic *Escherichia coli*	*K99*	GCTATTAGTGGTCATGGCACTGTAG	TTTGTTTTGGCTAGGCAGTCATTA	14
	Shigatoxigenic *Escherichia coli*	*stx1*	GACTGCAAAGACGTATGTAGATTCG	ATCTATCCCTCTGACATCAACTGC	15
	Shigatoxigenic *Escherichia coli*	*stx2*	ATTAACCACACCCCACCG	GTCATGGAAACCGTTGTCAC	15
	Shigatoxigenic *Escherichia coli*	*eaeA*	GACCCGGCACAAGCATAAGC	CCACCTGCAGCAACAAGAGG	16
	Enterohemorrhagic *Escherichia coli*	*hlyA*	GCATCATCAAGCGTACGTTCC	AATGAGCCAAGCTGGTTAAGCT	16
**Protozoa**	*Eimeria zuernii*	ITS-1	GCAAAAGTCGTAACACGGTTTCCG	CTGCAATTCACAATGCGTATCGC	17

Abbreviations: dsRNA segment 6, double-stranded RNA genome segment 6; VP, viral protein; N, nucleocapsid protein; S, S glycoprotein; M, membrane protein; RdRp, RNA-dependent RNA polymerase; MCP, major capsid protein; BstEII, restriction enzyme BstEII; *sefB*, chaperone protein SefB coding gene; *rfbJ*, CDP-abequose synthase coding gene; *K99*, K99 region 1 gene; *stx1*, shiga toxin type 1; *stx2*, shiga toxin type 2; *eaeA*, enterohemorrhagic *E. coli* O157:H7-specific intimin; *hlyA*, plasmid-encoded enterohemolysin; ITS-1, internal transcribed spacer 1 region of ribosomal RNA gene.

References

1. Chinsangaram, J., Akita, G.Y., Castro, A.E. & Osburn, B.I. PCR detection of group A bovine rotaviruses in feces. *Journal of veterinary diagnostic investigation : official publication of the American Association of Veterinary Laboratory Diagnosticians, Inc* **5**, 516-521 (1993).
2. Chang, K.O., Parwani, A.V. & Saif, L.J. The characterization of VP7 (G type) and VP4 (P type) genes of bovine group A rotaviruses from field samples using RT-PCR and RFLP analysis. *Archives of virology* **141**, 1727-1739 (1996).
3. Barman, P. et al. Sequencing and sequence analysis of VP7 and NSP5 genes reveal emergence of a new genotype of bovine group B rotaviruses in India. *Journal of clinical microbiology* **42**, 2816-2818 (2004).
4. Park, S.J. et al. Detection and characterization of bovine coronaviruses in fecal specimens of adult cattle with diarrhea during the warmer seasons. *Journal of clinical microbiology* **44**, 3178-3188 (2006).
5. Cho, K.O. et al. Cross-protection studies between respiratory and calf diarrhea and winter dysentery coronavirus strains in calves and RT-PCR and nested PCR for their detection. *Archives of virology* **146**, 2401-2419 (2001).
6. Duckmanton, L., Carman, S., Nagy, E. & Petric, M. Detection of bovine torovirus in fecal specimens of calves with diarrhea from Ontario farms. *Journal of clinical microbiology* **36**, 1266-1270 (1998).
7. Smiley, J.R., Hoet, A.E., Traven, M., Tsunemitsu, H. & Saif, L.J. Reverse transcription-PCR assays for detection of bovine enteric caliciviruses (BEC) and analysis of the genetic relationships among BEC and human caliciviruses. *Journal of clinical microbiology* **41**, 3089-3099 (2003).
8. Alkan, F. et al. Identification of a Bovine Enteric Calicivirus, Kirklareli Virus, Distantly

Related to Neboviruses, in Calves with Enteritis in Turkey. *Journal of clinical microbiology* **53**, 3614-3617 (2015).

9. Schmitt, B.J., Lopez, O.J., Ridpath, J.F., Galeota-Wheeler, J. & Osorio, F.A. Evaluation of PCR for diagnosis of bovine viral diarrhea virus in tissue homogenates. *Journal of veterinary diagnostic investigation : official publication of the American Association of Veterinary Laboratory Diagnosticians, Inc* **6**, 44-47 (1994).
10. Givens, M.D. et al. Analytical sensitivity of assays used for detection of bovine viral diarrhea virus in semen samples from the Southeastern United States. *Veterinary microbiology* **96**, 145-155 (2003).
11. Wu, J. et al. Detection and toxin typing of Clostridium perfringens in formalin-fixed, paraffin-embedded tissue samples by PCR. *Journal of clinical microbiology* **47**, 807-810 (2009).
12. Wang, S.J., Yeh, D.B. & Wei, C.I. Specific PCR Primers for the Identification of Salmonella enterica Serovar Enteritidis in Chicken-Related Samples. *J Food Drug Anal* **17**, 183-189 (2009).
13. Lim, Y.H. et al. Multiplex polymerase chain reaction assay for selective detection of Salmonella enterica serovar typhimurium. *Japanese journal of infectious diseases* **56**, 151-155 (2003).
14. West, D.M. et al. Rapid detection of Escherichia coli virulence factor genes using multiplex real-time TaqMan PCR assays. *Veterinary microbiology* **122**, 323-331 (2007).
15. Sharma, V.K. & Dean-Nystrom, E.A. Detection of enterohemorrhagic Escherichia coli O157:H7 by using a multiplex real-time PCR assay for genes encoding intimin and Shiga toxins. *Veterinary microbiology* **93**, 247-260 (2003).
16. Paton, A.W. & Paton, J.C. Detection and characterization of Shiga toxigenic Escherichia coli by using multiplex PCR assays for stx1, stx2, eaeA, enterohemorrhagic

E. coli hlyA, rfbO111, and rfbO157. *Journal of clinical microbiology* **36**, 598-602 (1998).

17. Kawahara, F. et al. Genetic analysis and development of species-specific PCR assays based on ITS-1 region of rRNA in bovine Eimeria parasites. *Vet Parasitol* **174**, 49-57 (2010).
