# Supplementary material for: Longitudinal evaluation of fecal microbiota transplantation for ameliorating calf diarrhea and improving growth performance
Source: Nat Commun. 2021 Jan 8;12:161. doi: 10.1038/s41467-020-20389-5 (PMC7794225; doi:10.1038/s41467-020-20389-5)
Supplement: Supplementary file 3 — Description of Additional Supplementary Files [file 41467_2020_20389_MOESM3_ESM.pdf]

Description of Additional Supplementary Files

File Name: Supplementary Data 1
Overview of the rectal 16S rRNA dataset generated using MiSeq during the preliminary trial.

File Name: Supplementary Data 2
Overview of the rectal 16S rRNA dataset generated using MiSeq during the validation trial.

File Name: Supplementary Data 3
Overview of the rectal 16S rRNA dataset generated using MiSeq for 12-month-old cattle.
