# Supplementary material for: Longitudinal evaluation of fecal microbiota transplantation for ameliorating calf diarrhea and improving growth performance
Source: Nat Commun. 2021 Jan 8;12:161. doi: 10.1038/s41467-020-20389-5 (PMC7794225; doi:10.1038/s41467-020-20389-5)
Supplement: Supplementary file 7 — Reporting Summary [file 41467_2020_20389_MOESM7_ESM.pdf]

nature research

Corresponding author(s): Jin-Woo Bae

Last updated by author(s): Nov 30, 2020

Reporting Summary

Nature Research wishes to improve the reproducibility of the work that we publish. This form provides structure for consistency and transparency in reporting. For further information on Nature Research policies, see our Editorial Policies and the Editorial Policy Checklist.

Statistics

For all statistical analyses, confirm that the following items are present in the figure legend, table legend, main text, or Methods section.

n/a	Confirmed	
☐	☒	The exact sample size (n) for each experimental group/condition, given as a discrete number and unit of measurement
☐	☒	A statement on whether measurements were taken from distinct samples or whether the same sample was measured repeatedly
☐	☒	The statistical test(s) used AND whether they are one- or two-sided<br>*Only common tests should be described solely by name; describe more complex techniques in the Methods section.*
☒	☐	A description of all covariates tested
☐	☒	A description of any assumptions or corrections, such as tests of normality and adjustment for multiple comparisons
☐	☒	A full description of the statistical parameters including central tendency (e.g. means) or other basic estimates (e.g. regression coefficient) AND variation (e.g. standard deviation) or associated estimates of uncertainty (e.g. confidence intervals)
☐	☒	For null hypothesis testing, the test statistic (e.g. F, t, r) with confidence intervals, effect sizes, degrees of freedom and P value noted<br>*Give P values as exact values whenever suitable.*
☒	☐	For Bayesian analysis, information on the choice of priors and Markov chain Monte Carlo settings
☐	☒	For hierarchical and complex designs, identification of the appropriate level for tests and full reporting of outcomes
☐	☒	Estimates of effect sizes (e.g. Cohen's d, Pearson's r), indicating how they were calculated

Our web collection on statistics for biologists contains articles on many of the points above.

Software and code

Policy information about availability of computer code

Data collection	Data collection and statistical analyses were performed using GraphPad Prism version 8.0 for Windows (GraphPad Software, La Jolla, CA, USA).

Data analysis

The adapter sequences were trimmed from the raw fastq files, and the trimmed reads were demultiplexed according to the samples using the bcl2fastq2 conversion software version 2.20.0. (Illumina).
The sorted reads were imported and processed using QIIME2 version 2020.06 for further bioinformatics analyses.
The imported paired reads were quality filtered, denoised, and merged using the plugin DADA2 (version 3.11) to generate the ASV feature table.
Taxonomic classification was performed using the plugin q2-feature-classifier, a taxonomic classifier plugin for the QIIME 2 microbiome analysis platform (https://qiime2.org/).
Taxonomy was assigned to filtered ASVs using a pre-trained QIIME2-compatible SILVA version 132 database, with 99% identity for the bacteria and representative sequences.
Treatment-dependent features were identified using LEfSe 1.0 (Biobakery/Vagrant VM).
The tool SourceTracker (version 1.0.1) was used to estimate the proportions of microbes from each source in a target microbial community (sink).
PICRUSt 1.1.4 (http://picrust.github.io), for predicting gene families based on the 16S rRNA gene composition, was used to characterize the functional profiles of the 12-month-old cattle gut microbial community.
The GC-TOF-MS data were acquired and pre-processed using LECO Chroma TOF™ software (version 4.44; LECO), and converted to NetCDF format (*.cdf) using LECO Chroma TOF™ software.
Peak detection, retention time correction, and alignment were performed using MetAlign 3.0 (http://www.metalign.nl), and the data were exported to an Excel file.
Statistical analyses were performed using GraphPad Prism version 8.0 for Windows (GraphPad Software, La Jolla, CA, USA).
Code used for generating the metataxonomic profiles is publicly available (DOI: 10.5281/zenodo.4176198, https://zenodo.org/record/4176198#.X59jiogzaUk, https://github.com/twwhon0111/FMT-treated-calf-gut-microbiome/).

For manuscripts utilizing custom algorithms or software that are central to the research but not yet described in published literature, software must be made available to editors and reviewers. We strongly encourage code deposition in a community repository (e.g. GitHub). See the Nature Research guidelines for submitting code & software for further information.

Data

Policy information about availability of data

All manuscripts must include a data availability statement. This statement should provide the following information, where applicable:

- Accession codes, unique identifiers, or web links for publicly available datasets
- A list of figures that have associated raw data
- A description of any restrictions on data availability

The raw sequences of the 16S rRNA genes obtained from the fecal, milk, and feed samples have been deposited in the European Nucleotide Archive, and are available under the accession number PRJEB35993 (https://www.ebi.ac.uk/ena/browser/view/PRJEB35993). The mass spectral raw data obtained from the fecal and serum samples have been deposited in the MetaboLights Workbench, and are available under the accession number MTBLS2226 (https://www.ebi.ac.uk/metabolights/MTBLS2226/). The source data underlying Figs. 1e, 2a-l, 3a-f, 4a-f, 5a-i and 6a-c, and Supplementary Figs. 1–9 are provided as a Source Data file.

Field-specific reporting

Please select the one below that is the best fit for your research. If you are not sure, read the appropriate sections before making your selection.

☒ Life sciences ☐ Behavioural & social sciences ☐ Ecological, evolutionary & environmental sciences

For a reference copy of the document with all sections, see nature.com/documents/nr-reporting-summary-flat.pdf

Life sciences study design

All studies must disclose on these points even when the disclosure is negative.

Sample size

Sample size was calculated by exceeding the minimum number of animals required for rarefaction. Basically, n ≥ 10 was chosen for the experiments comparing three groups, which sufficient to give a meaningful result to determine the effect of FMT. In the validation trial, fecal samples of diarrheic calves orally administered fecal microbiota (FMT, n = 20), saline buffer (CON, n = 14), or antibiotics (ABX, n = 23) were longitudinally collected 0, 2, 4, 8, 16, 32, and 48 days, and 12 months after treatment. A total of 450 (CON, n = 108; ABX, n = 176; FMT, n = 166) fresh fecal samples were used for the metataxonomic analaysis. The exact number of independent experiments was indicated in each figure legend.

Data exclusions

No data was excluded.

Replication

All attempts at replication were successful. In addition, the experiments which had been performed biologically in duplicate were described in Methods. The same treatment has been carried out in several animals to reinforce the results and to ensure the reproducibility of our results.

Randomization

Randomization was used for all biological experiments.
Calves with similar birth dates were randomly allocated to treatment groups, regardless of sex. Only healthy donor calves were chosen to participate in the validation trial.

Blinding

All analyses were carried out after fecal and serum sampling, which led to the setup of an experimental blinding procedure.
The investigators were blinded to the group allocation during sample collection.
For fecal microbial profiling, the investigators were blinded to the group allocation during DNA extraction and sequence data analysis. For Illumina Miseq 16S rRNA sequencing, the investigators were not blinded, but the sequencing was performed by different researchers.
For BCAA and amino acids quantification, the investigators were blinded to the group allocation during sample processing.

For metabolomics, the investigators were not blinded during sample preparation, metabolite extraction, metabolite profiling and data processing (GC-TOF-MS analysis), but the experiment was performed by different researchers.

Reporting for specific materials, systems and methods

We require information from authors about some types of materials, experimental systems and methods used in many studies. Here, indicate whether each material, system or method listed is relevant to your study. If you are not sure if a list item applies to your research, read the appropriate section before selecting a response.

Materials & experimental systems

n/a	Involved in the study
☒	☐ Antibodies
☒	☐ Eukaryotic cell lines
☒	☐ Palaeontology and archaeology
☐	☒ Animals and other organisms
☒	☐ Human research participants
☒	☐ Clinical data
☒	☐ Dual use research of concern

Methods

n/a	Involved in the study
☒	☐ ChIP-seq
☒	☐ Flow cytometry
☒	☐ MRI-based neuroimaging

Animals and other organisms

Policy information about studies involving animals; ARRIVE guidelines recommended for reporting animal research

Laboratory animals	The study did not involve laboratory animals
Wild animals	The study did not involve wild animals.
Field-collected samples	The study did not involve samples collected in the field.<br>The animals used in this study were Korean brown cattle (Bos taurus coreanae) raised on a farm located in Uiseong-gun, Gyeongsangbuk-do, Rebublic of Korea (36.372698, 128.540947), belonging to the National Korean Beef Association.<br>In the preliminary trial, 3 female and 4 male calves aged 9-38 days were used. In the validation trial, 3 female and 4 male calves aged 31-48 days were selected for donor calves. In the validation trial, 25 female and 32 male diarrheic calves aged 8-29 days were allocated for recipient calves.
Ethics oversight	The study protocol was approved by the Institutional Review Boards of the Kyung Hee University [KHUASP(SE)-17-028 and KHUASP(SE)-17-145] and complied with the Animal Protection Act and the Animal Welfare Guidelines of the World Animal Health Organization (WOAH, OIE).

Note that full information on the approval of the study protocol must also be provided in the manuscript.
